# Mapping the Dynamics of the Glucocorticoid Receptor within the Nuclear Landscape

**DOI:** 10.1038/s41598-017-06676-0

**Published:** 2017-07-24

**Authors:** Martin Stortz, Diego M. Presman, Luciana Bruno, Paolo Annibale, Maria V. Dansey, Gerardo Burton, Enrico Gratton, Adali Pecci, Valeria Levi

**Affiliations:** 1 Universidad de Buenos Aires, Facultad de Ciencias Exactas y Naturales, Buenos Aires, Argentina; 2 CONICET-Universidad de Buenos Aires, IFIBYNE, Buenos Aires, Argentina; 3 Laboratory of Receptor Biology and Gene Expression, National Cancer Institute, NIH, Bethesda, Maryland USA; 4 CONICET-Universidad de Buenos Aires, IFIBA, Buenos Aires, Argentina; 5 Max Delbrueck Center for Molecular Medicine, Berlin, Germany; 6 Universidad de Buenos Aires, Facultad de Ciencias Exactas y Naturales, Departamento de Química Biológica, Buenos Aires, Argentina; 7 CONICET-Universidad de Buenos Aires, UMYMFOR, Buenos Aires, Argentina; 8 Universidad de Buenos Aires, Facultad de Ciencias Exactas y Naturales, Departamento de Química Orgánica, Buenos Aires, Argentina; 9 Laboratory for Fluorescence Dynamics, Department of Biomedical Engineering, University of California, Irvine, USA; 10 CONICET-Universidad de Buenos Aires, IQUIBICEN, Buenos Aires, Argentina

## Abstract

The distribution of the transcription machinery among different sub-nuclear domains raises the question on how the architecture of the nucleus modulates the transcriptional response. Here, we used fluorescence fluctuation analyses to quantitatively explore the organization of the glucocorticoid receptor (GR) in the interphase nucleus of living cells. We found that this ligand-activated transcription factor diffuses within the nucleus and dynamically interacts with bodies enriched in the coregulator NCoA-2, DNA-dependent foci and chromatin targets. The distribution of the receptor among the nuclear compartments depends on NCoA-2 and the conformation of the receptor as assessed with synthetic ligands and GR mutants with impaired transcriptional abilities. Our results suggest that the partition of the receptor in different nuclear reservoirs ultimately regulates the concentration of receptor available for the interaction with specific targets, and thus has an impact on transcription regulation.

## Introduction

One of the most striking properties of nuclear processes is their compartmentalization. Many events do not occur homogeneously across the nucleus but are limited to defined spatial regions which are not physically separated by membranes^1, 2^. Different lines of research propose that some of these compartments concentrate specific proteins and RNAs involved in closely related processes and hence they increase the efficiency of reactions and facilitate their regulation^1^. However, this proposed functionality could not be generalized to every situation.

Particularly, many components of the transcription machinery ranging from transcription factors (TFs), coregulators, chromatin remodelers and RNA polymerases accumulate in distinct nuclear subdomains that dynamically exchange molecules with the nucleoplasm^3, 4^. Nuclear clusters formed by TFs do not always colocalize with regions of active transcription^5^, opening interesting questions regarding their biological function and how they assemble and disassemble.

In the present work, we explore the dynamic nuclear organization of the glucocorticoid receptor (GR), a ligand-activated TF that plays a relevant role in physiology^6, 7^. Steroid ligands bind to the predominantly cytoplasmic GR and upon activation the receptor translocates into the nucleus. Once in the nucleus, the GR regulates gene expression through direct binding to specific DNA regions named Glucocorticoid Response Elements (GREs)^8^, presumably as a tetramer^9^. Alternatively, it can modulate gene expression through interactions with other TFs^10^ either as a monomer or a dimer^11^. The transcriptional activity of GR depends on the local chromatin accessibility^12, 13^ and on specific coregulators. Activated GR preferentially associates with the nuclear receptor coactivator 2 (NCoA-2, also named GRIP1, SRC-2 or TIF2)^14, 15^. NCoA-2 is widely expressed in many tissues and is considered a primary GR cofactor due to its relevant role in the receptor response. It is recruited to GR-binding regions via a conserved receptor interaction domain in a ligand-dependent manner and facilitates transcriptional activation^16–18^ as well as transcriptional repression.

The activated GR forms a large number of nuclear foci, similarly to other TFs^19^. The biological relevance of these structures is still under debate^5, 20–23^. The partition of GR and its coregulators among the nucleoplasm and different nuclear bodies raises the question of how the receptor function is modulated by its localization and exchange rates between these compartments. A complete understanding of GR response requires integrating the transcriptional events occurring upon GR recruitment to DNA with the spatial and temporal GR distribution imposed by nuclear compartmentalization. Here, we address this question using a combination of advanced fluorescence microscopy techniques that are able to quantitatively determine the dynamics and interactions of GR in different nuclear compartments.

## Results

### Ligand-activation redistributes GR and NCoA-2 to colocalized intranuclear foci

Steroid receptors and its coactivators are heterogeneously distributed inside the nucleus upon stimulation with certain hormones^20, 23–26^. However, it remains poorly understood why or how this happens. To get insight into the molecular mechanisms behind this observation we used a combination of ligands and specific mutants of the GR. Specifically, we quantitatively characterized the organization of enhanced green fluorescent protein fused GR (GFP-GR) in BHK cells stimulated with dexamethasone (Dex), a selective and potent glucocorticoid receptor agonist or 21-hydroxy-6,19-epoxyprogesterone (21-OH), a dissociated GR agonist^6, 27^. We also explored the distribution of certain mutant forms of the receptor upon Dex stimulation: the GFP-GRmon, a monomeric receptor that does not efficiently interact with GREs^11^, and the GFP-GRdim, with impaired ability to directly induce gene expression^11, 28^.

**Figure 1:**
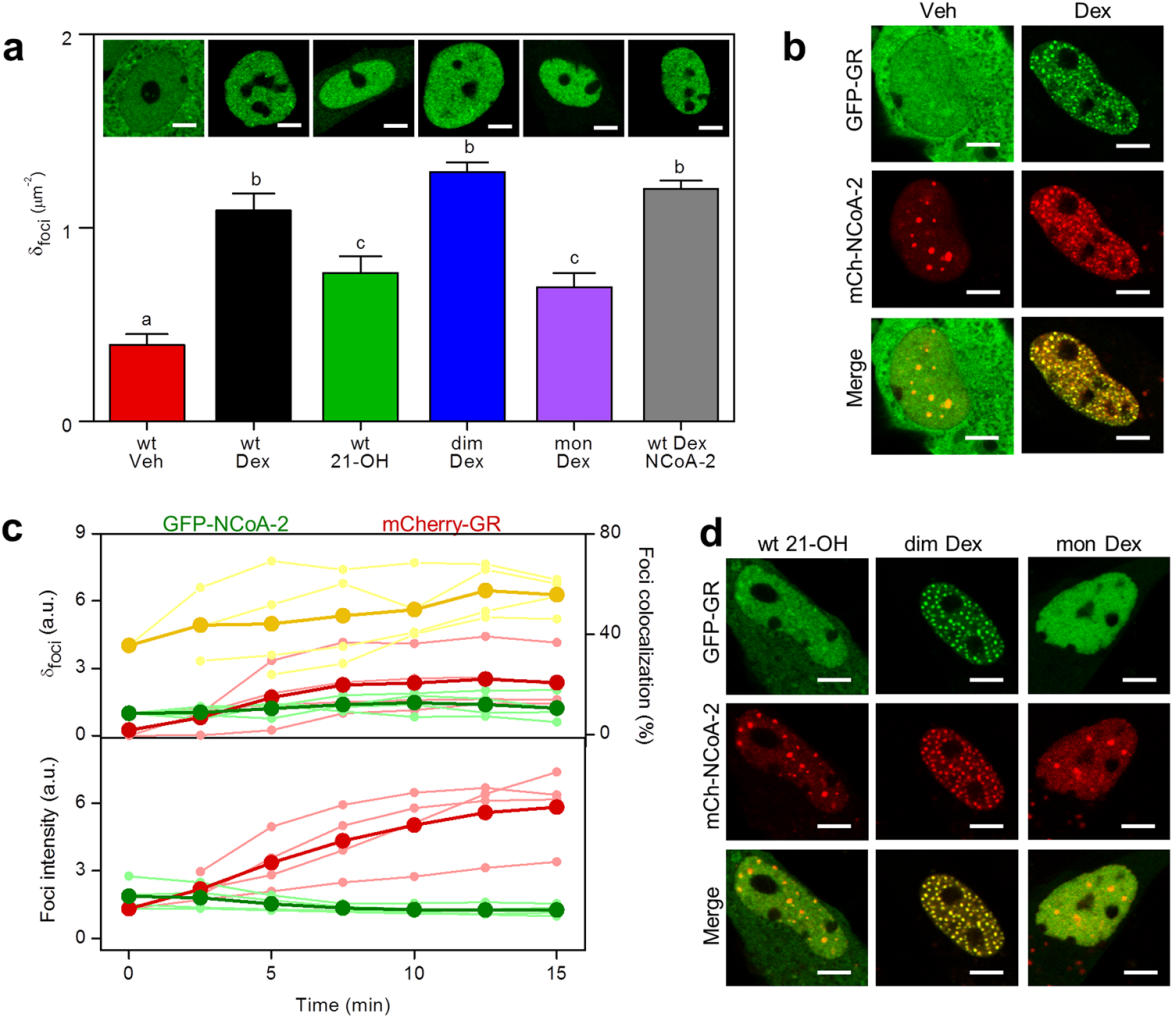
Activated GR and NCoA-2 redistribute to colocalized intranuclear foci. Subcellular distribution of GFP-GR and mCherry-NCoA-2 in BHK cells was analyzed upon hormone induction by using different imaging tools as indicated in Methods section. (**a**) Cells expressing GFP fused to the indicated GR variant (wild type, GRdim, GRmon) were treated with Dex, 21-OH or vehicle (Veh). NCoA-2 was co-expressed where indicated (Scale bar: 5 µm). GR capability to form intranuclear foci was quantitatively analyzed by calculating the nuclear foci density (n = 15). Bars with the same superscript letters represent data not significantly different from each other (p < 0.05). (**b**) Subcellular distribution of GFP-GR and mCherry-NCoA-2 in untreated or Dex-stimulated cells (Scale bar: 5 µm). (**c**) Analysis of time-lapse images of cells co-expressing GFP-NCoA-2 and mCherry-GR, within 15 min after Dex-stimulation. Different image-analyisis tools were used to calculate the relative foci density (δ) (upper panel); percentage of colocalized foci (upper panel, yellow line) and the relative mean intensity of foci (lower panel). Light curves represent single cell data to illustrate the cell-to-cell variability while dark curves correspond to mean values. (**d**) Representative images of cells expressing mCherry-NCoA-2 and GFP fused to the indicated GR variant (wild type, GRdim, GRmon) and treated with Dex, 21-OH or Veh (Scale bar: 5 µm).

Figure 1a shows that the intranuclear organization of GR depends on the specific hormone stimulation and receptor structure. We first evaluated the density of nuclear foci using image analysis tools and verified that Dex triggers the formation of a significantly higher number of foci in comparison with 21-OH (Fig. 1a) in line with our previous results^29^. On the other hand, Dex-stimulated GRdim (GRdim/Dex) distributed similarly to the wild type receptor whereas cells expressing GRmon stimulated with Dex (GRmon/Dex) presented a significantly smaller density of foci (Fig. 1a).

We next explored how the coactivator NCoA-2 modulates the nuclear distribution of GR in BHK cells co-transfected with GFP-GR and mCherry-NCoA-2 (Fig. 1b). In non-stimulated cells, the coactivator formed nuclear bodies that colocalized with promyelocytic leukemia (PML) protein fused to GFP (Supplementary Fig. S1a) in line with previous results from Baumman *et al*.^25^ who identified PML bodies by indirect immunofluorescence and observed colocalization with GFP-NCoA-2 bodies. These authors also showed that neither the amount of transfected GFP-NCoA-2 expression vector nor the total fluorescence intensity of an individual cell affect bodies formation suggesting that focal accumulation of GFP-NCoA-2 is not an artifact of overexpression; this hypothesis should be further tested by immunolabeling the endogenous NCoA-2.

NCoA-2 redistributed to numerous small and dimmer foci upon Dex stimulation (Fig. 1b) in a reversible manner (Supplementary Fig. S1b). The co-expression of NCoA-2 did not affect the density of GR nuclear foci (Fig. 1a). The ligand dependent redistribution of NCoA-2 and the reversibility of this process suggest that NCoA-2- bodies are not consequence of an unspecific aggregation of overexpressed proteins.

The reorganization of both GR and NCoA-2 was then followed by time-lapse confocal imaging. The nuclear intensity of mCherry-GR increased with time as expected from the translocation of the receptor into the nucleus upon receptor activation while GFP-NCoA-2 intensity remained constant (Supplementary Fig. S2). The density of mCherry-GR foci also increased and reached a plateau after ~8 min (Fig. 1c). GR foci kept incorporating receptor molecules, as assessed from the increment on their mean intensity. The colocalization between the receptor and cofactor foci also increased with time suggesting a continuous redistribution of these molecules to colocalizing foci.

The nuclear reorganization of NCoA-2 upon hormone stimulation depends on the ligand since GR stimulated with 21-OH (GR/21-OH) was partially recruited to the NCoA-2- bodies but did not trigger the formation of small NCoA-2 containing foci (Fig. 1d). This result supports our previous work showing that GR/21-OH does not co-precipitate with NCoA-2^29^. In addition, GRmon/Dex was also unable to cause NCoA-2 redistribution whereas GRdim/Dex showed a similar distribution to that observed with the Dex-stimulated wild type receptor (GR/Dex).

### GR and NCoA-2 dynamically partition between the nucleoplasm and DNA-dependent discrete foci

**Figure 2:**
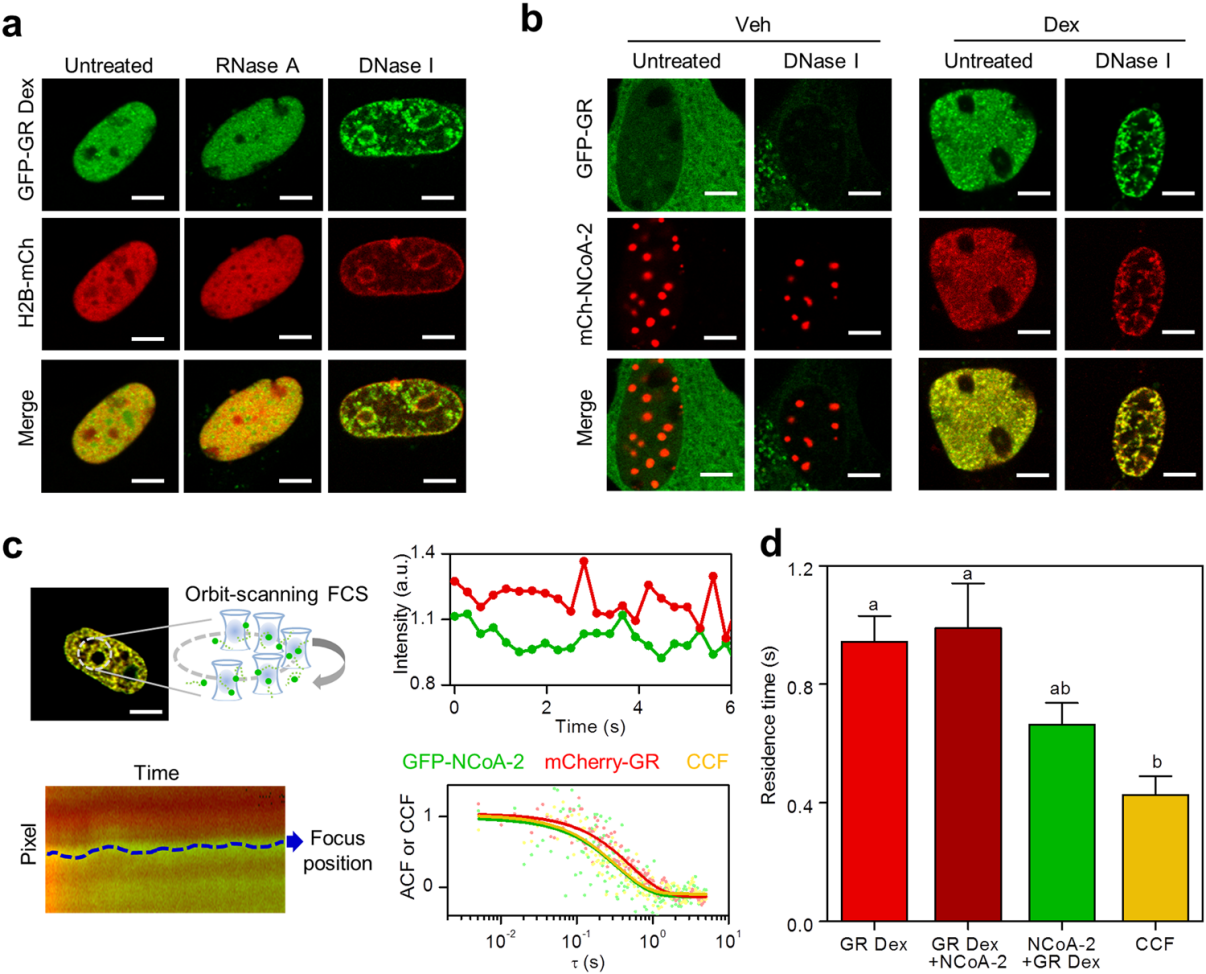
GR and NCoA-2 dynamically partition between the nucleoplasm and DNA-dependent discrete foci. Properties of GR nuclear foci in BHK cells were analyzed using biochemical and FCS-based approaches. (**a**) Cells co-expressing GFP-GR and H2B-mCherry were fixed, permeabilized and digested with RNase A or DNase I (Scale bar: 5 µm). (**b**) *In situ*-DNase I digestion of cells co-expressing GFP-GR and mCherry-NCoA-2 before and after stimulation with Dex (Scale bar: 5 µm). (**c**,**d**) Orbit-scanning FCS experiments were run in cells expressing either GFP-GR or GFP-NCoA-2 and mCherry-GR. (**c**) The laser orbits were set to scan through foci which position were further determined with subpixel precision. The intensity traces of foci were then used to calculate the autocorrelation or cross-correlation functions (representative curves). ACF data was fitted with a model that considers binding to fixed targets (continuous lines). (**d**) Mean residence time of mCherry-GR (dark red), GFP-NCoA-2 (green) and CCF (yellow) at foci (n = 12). Red bar corresponds to the residence time of GFP-GR in cells that were not cotransfected with NCoA-2. Bars with the same superscript letters represent data not significantly different from each other (p < 0.05).

In order to assess the nature of these GR nuclear foci, we cotransfected BHK cells with GFP-GR and H2B-mCherry as a marker of DNA integrity. After Dex stimulation, we performed *in situ* nuclease-digestion experiments. Deoxyribonuclease I (DNase I) but not ribonuclease A (RNase A) treatment severely compromised the formation of GR foci (Fig. 2a). GFP-GR in DNA-digested cells appears to be mostly localized in perinuclear and perinucleolar regions, probably containing highly-condensed chromatin inaccessible to DNase I action. The coregulator distribution was similarly affected by DNase I digestion as assessed in Dex-stimulated BHK cells co-expressing GFP-GR and mCherry-NCoA-2 (Fig. 2b). Taken together, these results suggest that foci formation relies on the integrity of DNA.

We next explored the exchange of GR and NCoA-2 molecules at foci using a two-color fluorescence correlation spectroscopy (FCS) approach. FCS methods analyze intensity fluctuations caused by fluorescent molecules moving through the small observation volume of a confocal or two-photon excitation microscope^30–32^. Through a correlation analysis of these fluctuations, FCS provides quantitative information on the dynamics of the molecules. Due to its non-invasive character, FCS is considered a useful tool to study the dynamics of transcription factors both in culture cells^33, 34^ and *in vivo* ^35–38^.

Single-point FCS approaches failed to recover the dynamics of the receptor and its coactivator at the foci since their slow translational motion introduces artifacts in the measurements (Supplementary Fig. S3). To correct for this motion, we used a laser scanning approach^30^. Specifically, we set the microscope to continuously scan the laser along an orbit and obtained an intensity kymograph which was further analyzed with a line-tracking algorithm to position the center of the foci with subpixel precision^39, 40^. We then recovered the intensity at the focus as a function of time and performed an autocorrelation analysis (Fig. 2c). The autocorrelation function (ACF) data of both mCherry-GR and GFP-NCoA-2 could be described with a model that considers the slow binding of these proteins to fixed targets^41^. The orbit sampling time (5.1 ms) was relatively slow in comparison to the characteristic diffusion time of GR and NCoA-2 in the nucleoplasm (Supplementary Fig. S4). Thus, this approach does not allow us to observe freely diffusing molecules. The mean residence time at these targets ranged from 0.5 to 1 s (Fig. 2d), indicating that these DNA-dependent foci are not static repositories but rather have a dynamic exchange of molecules with the nucleoplasm.

We also performed a cross-correlation analysis of the mCherry-GR and GFP-NCoA-2 intensity traces. This method detects hetero-complexes formed by molecules labeled with different fluorescent probes quantifying correlated fluctuations in the intensity traces obtained for the probes^31^. A positive cross-correlation at the foci (Fig. 2c) suggests that binding of GR and NCoA-2 are not independent from each other. Moreover, the cross-correlation lifetime was significantly smaller than the residence time of GR (Fig. 2d), suggesting that these proteins sequentially bind to the foci (e.g. GR interacts with the focus and then NCoA-2 is recruited) and/or sequentially unbind from it (e.g. NCoA-2 and GR detach one after the other).

### GR conformation influences the dynamic recruitment to an array of GREs

**Figure 3:**
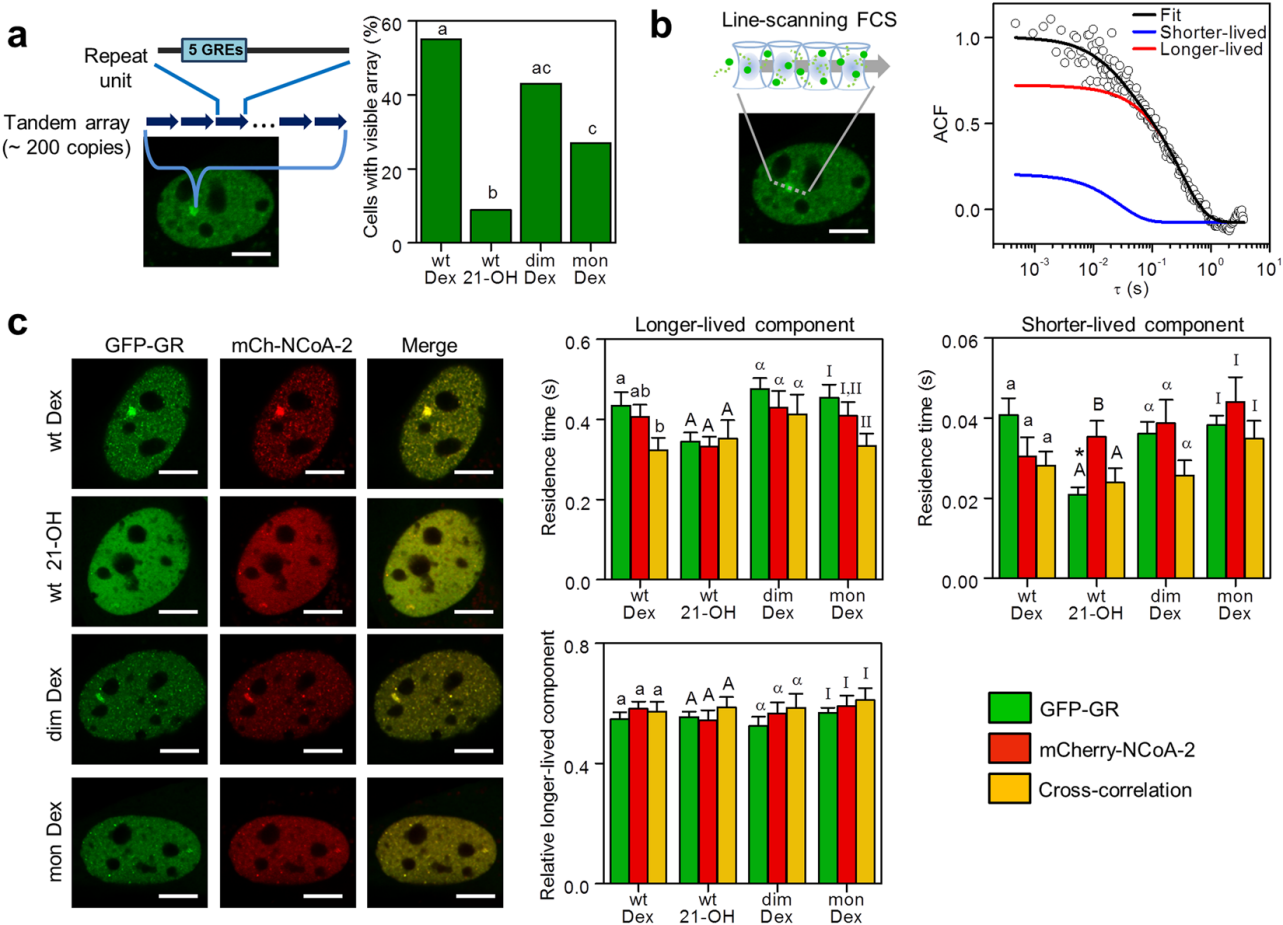
GR and NCoA-2 interactions with GREs depend on the receptor conformation. (**a**) Representative image of a 3617 cell expressing GFP-GR. The MMTV-array is visualized as a bright spot in the nucleus (Scale bar: 5 µm). The ability of GR recruitment to the MMTV-array was analyzed in cells expressing the receptor fused to GFP (wild type, GRdim or GRmon) and treated with Dex or 21-OH as the percentage of cells showing a visible array (69 < n < 79). Bars with the same superscript letters represent data not significantly different from each other (p < 0.05) according to Marascuilo’s multiple comparisons procedure (see Methods). (**b**) Line-scanning FCS experiments were run in cells expressing GFP-GR; the laser was set to scan along a line across the MMTV-array which position was then determined with subpixel precision to obtain the intensity time-trace. Representative ACF data fitted with a model that considers binding to fixed targets in two different time-scales (equation (3), continuous line). (**c**) Similar line-FCS experiments run in cells expressing mCherry-NCoA-2 and GFP-GR (wild type, GRdim or GRmon), and treated with Dex, 21-OH or vehicle (Veh) (22 < n < 26) (Scale bar: 5 µm) provided the mean residence times at shorter-lived and longer-lived targets and the relative proportion of molecules bound to longer-lived sites with respect to those engaged in interactions. Bars with the same superscript letters and text font represent data not significantly different from each other (p < 0.05). Bars marked with asterisk (*) represent data significantly different from those obtained in the control condition (GFP-GR + mCherry-NCoA-2 + Dex) (p < 0.05).

We next analyzed the interactions of GR with DNA enriched in specific response elements using the 3617 cell line. These cells present a large tandem array (~200 copies) of a mouse mammary tumor virus, Harvey viral ras (MMTV-v-Ha-ras) reporter that contains 5 GREs in each copy (Fig. 3a)^42–44^. The tandem array is easily detected in GFP-GR transfected cells stimulated with Dex as a bright spot in the nucleus^45^, although with great cell-to-cell variability in terms of the size of the array^46^. We thus only evaluated the ability of the receptor to be recruited to this region by determining the fraction of cells showing detectable recruitment to the array (p_array_). These fractions were similar for GR/Dex and GRdim/Dex. However, GR/21-OH and GRmon/Dex presented impaired ability to generate arrays in line with our previous studies^11^.

To explore the binding dynamics of the wild type receptor to the MMTV-array, we followed a line-scanning FCS approach (Fig. 3b) to correct for the slow motion of the array, similarly to the orbit-scanning method described above. The time resolution of this line-FCS approach (0.76 ms) allowed capturing the binding dynamics of the GR at the MMTV-array.

Supplementary Fig. S5 shows that this data could not be explained by considering GR recruitment to a population of identical binding sites. This result was expected since the femtoliter-sized volume sampled through FCS probably includes the MMTV-array and other chromatin loops. In a previous work^35^, we had observed that TF dynamics in nuclei of early mouse embryos could be explained considering TF/DNA binding events in two different time scales. Thus, we followed a similar reasoning and included two populations of binding sites with distinct kinetics to account for the slower and faster interactions (equation (3)). Figure 3b and Supplementary Fig. S5 showed that this model could describe the ACF data.

We next assayed the interactions of GR and NcoA-2 at the MMTV-array using dual color line-FCS in 3617 cells expressing mCherry-NCoA-2 and GR or the mutant receptors fused to GFP and presenting a visible array (Fig. 3c). The longer-lived component represented ~55% of the bound population and its residence time ranged in 350–500 ms, while the lifetime of the shorter-lived interactions was 20–45 ms (Fig. 3c).

The cross-correlation analysis showed that GR/Dex and NCoA-2 interact within the MMTV-array (Fig. 3c and Supplementary Fig. S6). The longer-lived residence time of the Dex-activated receptor was significantly higher than that of the cross-correlation suggesting that the receptor and its coactivator interact sequentially with the targets.

The binding properties of the mutant receptors at the MMTV-array were similar to those observed for the wild type receptor. In contrast, GR/21OH presented shorter residence times, probably reflecting an impaired binding ability of this activated form of the receptor (Fig. 3c).

### Fluorescence correlation spectroscopy reveals different landscapes of GR/NCoA-2 nuclear interactions

**Figure 4:**
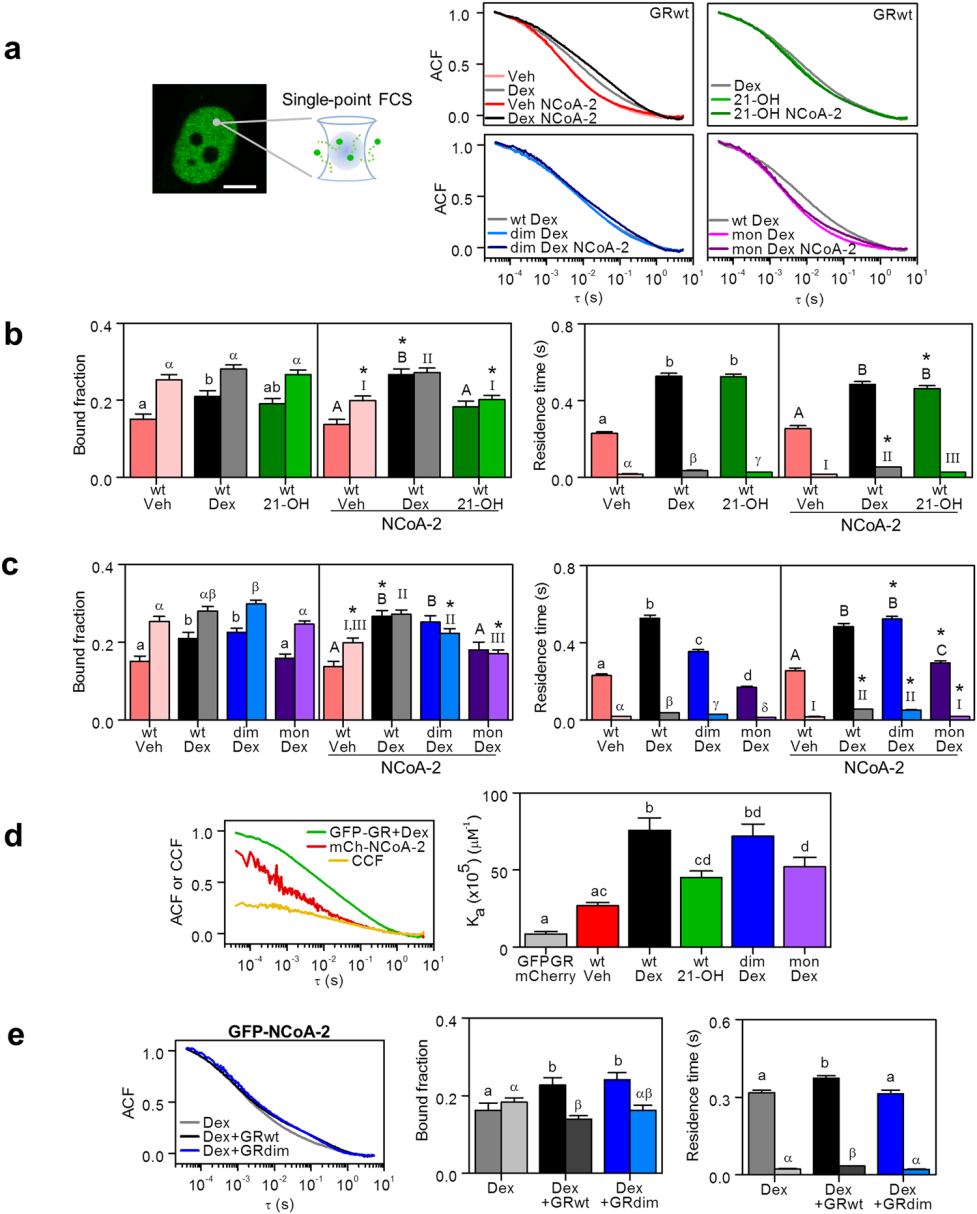
Fluorescence correlation spectroscopy reveals different landscapes of GR/NCoA-2 nuclear interactions. Single-point FCS measurements were run in the nucleoplasm of BHK living cells expressing GFP fused to the wild type GR, GRdim or GRmon and treated with Dex, 21-OH or vehicle (Veh), in the absence or presence of mCherry-NCoA-2. (**a**) Average normalized ACF of the different GFP-GR variants in the specified conditions. (**b**,**c**) ACF data was fitted with equation (2) to obtain the bound fraction of receptors and residence times engaged in shorter-lived (light colors) and longer-lived (dark colors) interactions (25 < n < 38). Asterisks (*) indicate that a parameter significantly differs due to mCherry-NCoA-2 overxpression (p < 0.05). For parameters measured in the absence of mCherry-NCoA-2, lower case (a, b, c) or Greek (α, β, γ) letters indicate significant differences among longer- or shorter-lived components, respectively (p < 0.05). For parameters measured in the presence of mCherry-NCoA-2, upper case letters (A, B, C) or Roman numbers (I, II, III) indicate signifcant differences among longer- or shorter-lived components, respectively (p < 0.05). (**d**) The apparent association constant (K_a_) between the GFP-tagged receptor (wild type, GRdim or GRmon) and mCherry-NCoA-2 in cells treated with Dex, 21-OH or Veh was calculated from the ACFs and CCF amplitudes (20 < n < 25). Bars with the same superscript letters represent data not significantly different from each other (p < 0.05). Control condition: GFP-GR and mCherry co-expressed in Dex-stimulated cells. Left panel shows the mean, normalized ACFs of GFP-GR and mCherry-NCoA-2 and CCF in cells stimulated with Dex. (**e**) Average normalized ACFs of GFP-NCoA-2 in Dex-stimulated cells, in the absence or presence of mCherry-GR (wild type or GRdim). The ACF data was fitted with equation (2) to obtain the bound fraction of GFP-NCoA-2 engaged in interactions with fixed targets and the shorter-lived (light colors) and longer-lived (dark colors) residence times (22 < n < 43). Lower case (**a**,**b**) or Greek (α, β) letters indicate significant differences among longer- or shorter-lived components, respectively (p < 0.05).

We next used single-point FCS to measure the local dynamics of GR and its coactivator in the nucleoplasm. The overall motion of GFP-GR in the nucleus of BHK cells is considerably slower than GFP alone (Supplementary Fig. S7), reflecting different landscapes of interactions in the nucleus. Stimulation with Dex shifted GR dynamics to longer time lags (Fig. 4a) as expected from the activation of the receptor. Supplementary Fig. 7 shows that the ACF of GFP-GR could not be fitted considering only the diffusion and binding of the receptor to a single population of fixed targets^47, 48^. In line with the FCS observations in the 3617 cell line, we included in the model interactions with targets in two distinct temporal windows (equation (2)). This model could correctly fit the experimental results (Supplementary Fig. S7).

The fraction of GR/Dex molecules bound to longer-lived sites was smaller in single-point FCS experiments (Fig. 4b,c) than at the MMTV-array (Fig. 3c). Indeed, this was an expected result since the nuclear volume sampled in the MMTV experiments probably contains a larger proportion of specific, longer-lived binding sites in comparison to the volume sampled in single-point FCS measurements. Thus, the longer-lived fraction is expected to be smaller in these last measurements. On the other hand, the residence time of GR/Dex of both bound populations were similar to those measured at the MMTV-array.

Figure 4a,b shows that both the fraction of GR bound to the longer-lived sites and its mean lifetime are similar upon Dex or 21-OH stimulation.

The distribution of GRdim/Dex was similar to that observed with the wild type receptor whereas the interactions at longer-lived sites were faster (Fig. 4a and c). In contrast, the bound fraction of GRmon/Dex was similar to that observed in non-stimulated cells whereas the longer lifetimes were slightly smaller (Fig. 4a and c), probably reflecting the impaired ability of this mutant to interact with certain DNA sites^11^. ACF curves obtained in non-stimulated cells expressing GFP-GRdim or GFP-GRmon were similar to that observed for the wild type receptor (Supplementary Fig. S8).

We next assessed whether the coactivator NCoA-2 modulates GR dynamics. Stimulation with Dex increased the bound fraction but not the lifetime of GR molecules attached to the longer-lived sites with respect to those observed in the absence of mCherry-NCoA-2, suggesting that increasing the coactivator levels facilitates the interaction of GR with longer-lived sites (Fig. 4a,b). In contrast, mCherry-NCoA-2 expression did not modify the bound fraction and lifetime of GR/21-OH at the longer-lived sites whereas it promoted the detachment of the receptor from the shorter-lived sites (Fig. 4a,b). NCoA-2 also decreased the fraction of receptor at the shorter-lived sites in non-stimulated cells and in Dex-stimulated cells expressing the mutant forms of the receptors GRdim and GRmon. In the case of GRdim, we also verified that NCoA-2 increased the mean lifetime of the receptor at the longer-lived sites (Fig. 4a and c). Taken together, these results suggest that GR-NCoA-2 interactions modulate the distribution of GR among different nuclear targets. This redistribution seems to be finely tuned by the receptor conformation.

Next, we tested whether GFP-GR and mCherry-NCoA-2 could interact in the nucleoplasm of BHK cells using a cross-correlation analysis. The positive cross-correlation evidences the formation of GFP-GR/mCherry-NCoA-2 complexes in the nuclei of living cells (Fig. 4d). Unfortunately, we could not analyze quantitatively the cross-correlation curves with equation (2) (see Methods section) due to the higher noise in these curves with respect to the ACF curves. Thus, we computed an apparent equilibrium association constant K_a_ (equation (1)) to quantify the relative proportion of associated GR/NCoA-2 molecules (Fig. 4d). Dex increased the association between GR and its coactivator as previously reported^15^. In contrast, stimulation with 21-OH resulted in a lower proportion of heterocomplexes since this ligand does not efficiently allow the GR/NCoA-2 interaction (Fig. 1d)^29^. GRdim/NCoA-2 showed a similar value of K_a_ with respect to the wild type receptor whereas GRmon showed an impaired interaction with the coactivator as assessed by the low K_a_ value determined in this condition.

The analysis of the ACF curves of GFP-NCoA-2 in the presence of GR/Dex showed that the stimulated receptor increased both the residence time and the bound fraction of NCoA-2 at longer-lived sites (Fig. 4e). In contrast, this lifetime did not change when the GRdim mutant was co-expressed suggesting that the mutation introduced in the receptor impairs the interaction with the coactivator at longer-lived sites. The population fraction of GFP-NCoA-2 at longer-lived sites was similar in both conditions.

Supplementary Fig. S9 details the results obtained for shorter-lived interactions.

## Discussion

The puzzling organization of the mammalian nucleus is gaining attention for its relevance in many cellular processes. Numerous studies have identified a growing number of domains, foci or nuclear substructures with no membranes separating them from the nucleoplasm. The hierarchical and dynamical architecture of chromatin also imposes a higher order of regulation to the organization of nuclear components^4^.

The glucocorticoid receptor has attracted the attention of many researchers due to its pharmacological relevance^49^. GR dynamics was previously explored *in vivo* through different fluorescence microscopy methods such as fluorescence recovery after photobleaching^50^, single point fluorescence correlation spectroscopy^48^ and single molecule tracking^47, 51, 52^. These previous studies focused either on the global mobility of the receptor or on its interaction with nuclear targets and thus could not provide clues on the overall dynamical organization of the GR in the nuclear compartment.

In this work, we used a combination of fluorescence fluctuation-based methods to explore how the GR and its coactivator NCoA-2 are organized in the nucleus and how their distributions vary in response to certain stimuli.

FCS studies require low but significant levels of fluorescent protein expression to obtain an S/N ratio adequate for the measurements. This is the main reason of why most of live cell microscopy studies rely on cells overexpressing fluorescent-tagged proteins. In this direction, we and others verified that many biological functions of the endogenous GR are preserved in the fluorescent receptor (e.g. transcriptional activity^11, 29, 53, 54^, oligomerization state^11^, spatial distribution^23, 53^). These properties depend on the ligand and are reversible by washout experiments^11, 55^ as also expected for the endogenous receptor.

**Figure 5:**
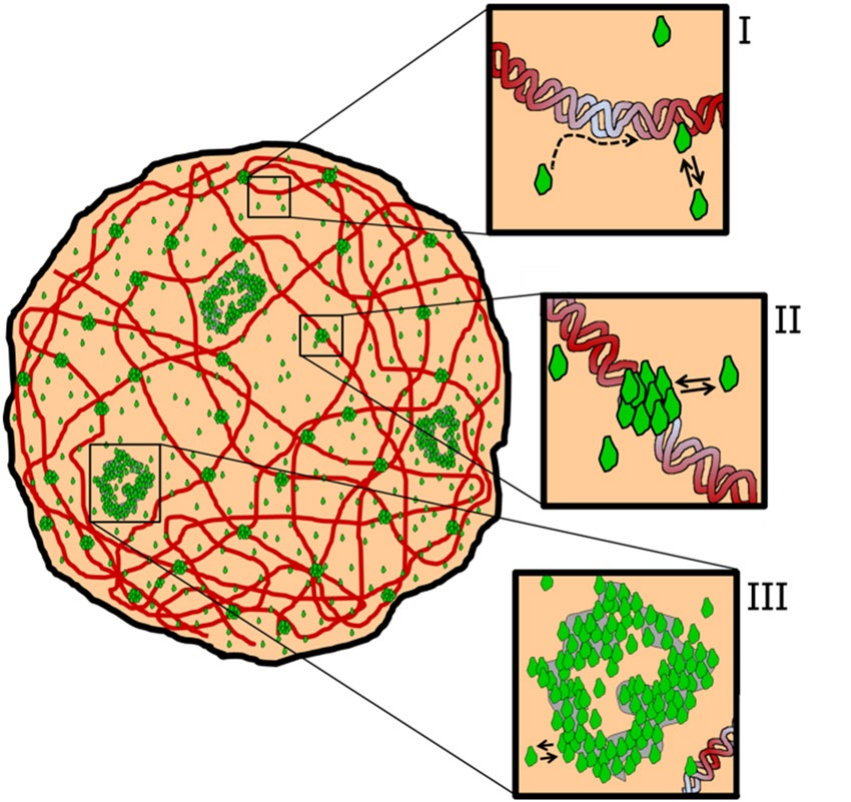
Model of the dynamic nuclear organization of GR. Schematic representation of GR dynamic partition within the nuclear landscape. GR diffusing molecules engage in shorter-lived and longer-lived interactions with chromatin targets (I), DNA-dependent foci (II) or NCoA-2 bodies (III). To simplify the scheme, the cartoon does not include the oligomeric state of GR.

Figure 5 shows a schematic drawing describing some aspects of the functional partitioning of the glucocorticoid receptor. GR molecules diffuse within the nucleus and may interact with a variety of chromatin targets (I), DNA-dependent foci (II) and NCoA-2 bodies (III). The relative proportion of receptor molecules engaged in either interaction depends on the ligand-dependent conformation of the receptor and the presence of coregulators. In this work, we used conservative and somewhat arbitrary definitions of chromatin targets and foci based on our ability to detect them (which is proportional to the number of recruited TF molecules). We consider “chromatin targets” as those involved in the interaction with small TF complexes with low and defined stoichiometry (e.g. ref. 9) unresolvable with standard confocal microscopy. In contrast, we consider “DNA-dependent foci” as those structures formed by the dynamical recruitment of a large number of TF molecules with an apparently variable stoichiometry.

Whereas it is not completely clear the biological relevance of TF clusters, growing evidence indicates that they may be an important mode of regulation of transcription^56^. GR foci form in the endogenous background^5, 23^ and their formation is modulated by ligands^55^ also stressing their biological relevance. Single-point FCS measurements showed that the dynamics of the glucocorticoid receptor in the nucleoplasm could be explained by a model that considers the diffusion of GR and binding in two different time-scales (Fig. 4a–c). GR-chromatin interactions may include non-specific binding, short-distance sliding on DNA, hopping^57–61^ or long-lived and more specific binding^51^. We hypothesize that shorter-lived interactions are probably involved in the searching process required to find specific targets in the complex nuclear environment^62, 63^. Thus, the impairment of this fast-searching step may affect the capability of the receptor to efficiently locate its specific binding sites. Moreover, the interplay between receptor conformation and local chromatin architecture and plasticity seems to be very relevant on this functional partition as assessed from previous observations^11, 12, 64^.

According to our FCS data from the nucleoplasm, the free, diffusing fraction represents 45–65% whereas the remaining population of the total GFP-GR molecules is engaged in shorter-lived and longer-lived interactions (Fig. 4b,c). These proportions vary with ligand and appear to depend on the receptor conformation. The lifetimes of shorter and longer-lived interactions ranged 20–60 ms and 200–500 ms, respectively (Fig. 4b,c). The temporal window of interactions observed at the MMTV-array was in the same time scale (Fig. 3c). In this sense, the longer-lived component observed in this work may represent fast specific binding events.

Recently, single molecule tracking (SMT) methods detected GR binding events exponentially distributed in a longer time scale, with a characteristic dwell time between 2–8 seconds^47, 51, 52, 65^. These different results are probably due to the time windows explored with either method; whereas SMT usually requires low acquisition frequencies in the order of 1–100 Hz and can detect slow and less-frequent interactions, FCS samples at a much higher speed (50000 Hz) and therefore collects information on faster interactions. In addition, the residence times at GR binding sites are expected to span a wide temporal window since they depend on different properties of the microenvironment such as the local structure of the chromatin^51^.

In this work we considered GR diffusion to be fast compared to the binding kinetics as assumed in the reaction-dominant approximation proposed by Michelman *et al*.^41^, also used to describe protein-DNA interactions in cells. We are aware that the autocorrelation analysis is not able to discriminate the binding and unbinding probabilities at targets unless assumptions are made in regard to the binding probability. Unfortunately, this probability depends on unknown local parameters such as the number of binding sites, the concentration of GR and its diffusion coefficient. Under our approximation, the diffusion process is relatively unimportant at the time scale observed for GR interactions with the nuclear targets.

Biological processes involved in gene expression take place in the extremely crowded and complex nuclear environment where regulation mechanisms operate at different levels of complexity^66^. TF function relies on the conformation of the biomolecule, its searching strategies in the tortuous nuclear space and the global and local architecture of chromatin exposing or hiding surfaces with different affinities. Fluorescence fluctuation approaches with spatial correlation analyses permitted exploring the intricate topology of the nucleus with unprecedented resolution^67–69^. Particularly, Baum *et al*.^69^ concluded that the intranuclear milieu behaves as a multi-scale porous medium formed by randomly distributed obstacles that constrain the diffusion of inert molecules in a size-dependent manner. These authors showed that the behavior of the tracers is well described by two normal diffusion modes characterized by a microscopic and macroscopic diffusion coefficients connected by a crossover region. Inert molecules of dimensions similar to that of the activated GR are expected to explore the confocal observation volume in less than 20–40 ms^69, 70^ with a crossover between these microscopic and macroscopic regimes occurring at distances larger than the observation volume^69^.

On the other hand, chromatin-interacting proteins present delayed dynamics in comparison to inert tracers^69^ reflecting fast interactions with nuclear targets. In addition, single particle tracking methods reported that the exploration geometry of TFs is mainly restrained by their interactions with nuclear structures and not by exclusion^71^.

Taken together, these antecedents suggest that the dynamics of GR and its coactivator in the millisecond-time scale may probably be affected by the complex architecture of the nucleus. However, the dependence of the shorter-lived component on the receptor conformation and NCoA-2 association (Fig. 4a–c) cannot be explained by only considering the anomalous diffusion of inert molecules of different sizes, but rather requires the inclusion of fast interactions of both receptor and coactivator with nuclear targets.

DNA-dependent foci (Fig. 2a,b) dynamically capture receptor and coactivator molecules which do not interact with targets as stable complexes. Instead, our data suggest that these molecules bind or unbind sequentially from foci (Fig. 2c,d). We propose that the dynamical nature of foci may constitute an additional mechanism to regulate the effective concentration of the receptor and coactivator in the nucleoplasm and may indirectly affect transcription. In the same direction, the interaction of NCoA-2 with PML nuclear bodies was also proposed to modulate the coactivator availability^25, 72^.

We also found that the dynamic distributions of GR/21-OH and GRmon/Dex complexes presented some common features. Specifically, these receptor/ligand complexes translocate into the nucleus but do not associate efficiently to the coactivator (Fig. 4d) or trigger NCoA-2 redistribution to DNA-dependent foci (Fig. 1d). These results suggest that foci formation depends on the capability of the receptor to interact with NCoA-2. The coactivator also promoted the detachment of both activated receptor complexes from shorter-lived sites (Fig. 4b,c). Even though GR/21-OH and GRmon/Dex may interact with the MMTV-array, the proportion of cells presenting the characteristic bright spot was small in both cases (Fig. 3a), reflecting their impaired ability to induce MMTV-triggered gene expression^11, 29^. The mechanisms involved in these deficient inductions seem to be different: whereas GR/21-OH presented a high detachment rate (i.e. k_off_) from the MMTV-array, GRmon/Dex unbinding rate was similar to that observed for GR/Dex, suggesting that the impaired interaction is a consequence of a low attachment probability (Fig. 3c). These conformations also present some differences on their partition landscapes in the absence of mCherry-NCoA-2. Particularly, GRmon/Dex binding was severely compromised since its performance was similar to that observed for the non-stimulated receptor (Fig. 4b,c). This behavior is likely related to the impaired functionality of this monomeric mutant^11^.

On the other hand, the GRdim/Dex spatial distribution, its affinity to NCoA-2 and its dynamics at binding sites resemble those observed for GR/Dex. The main differences between this mutant and the wild type receptor appear to be its lifetime at longer-lived sites and its NCoA-2-dependent redistribution. Particularly, the coactivator increases the longer-lived population of GR/Dex but promotes the detachment of GRdim/Dex from shorter-lived sites similarly to GR/21-OH and GRmon/Dex (Fig. 4d). Moreover, the dim mutation affects the residence time of the coactivator at longer-lived targets. This impaired interaction may be related to the inability of this mutant to upregulate gene expression. Further work is required to determine whether the inability of GRdim to be allosterically modulated by DNA^73^ influences GRdim-NCoA-2 interaction.

In conclusion, we quantitatively described the highly dynamic and heterogeneous distribution of GR and its coactivator NCoA-2 in the cell nucleus and presented evidence demonstrating that these distributions are finely tuned by the receptor conformation. This new insight contributes in deciphering how the complex landscape of the interphase nucleus impacts gene expression.

## Methods

### Steroids and reagents

Dex, RNase A and DNase I were purchased from Sigma-Aldrich (St. Louis, MO, USA). 21-OH was prepared as described^74^. Dulbecco’s modified Eagle’s medium (DMEM) and Lipofectamine 2000 were purchased from Thermo Fisher Scientific (Waltham, MA, USA). Fetal bovine serum (FBS) was purchased from Internegocios (Mercedes, Buenos Aires, Argentina).

### Plasmid constructs

pEGFP-GR^A465T^ (GRdim) and pEGFP-GR^A465T/I634A^ (GRmon) were previously described^11^. pEGFP-GR, pEGFP-NCoA2, pEGFP-PML-I and pH2B-mCherry were kindly provided by Mario Galigniana^53^, Monica Costas^75^, Peter Hemmerich^76^ and Robert Benezra (Addgene plasmid #20972)^77^, respectively. pmCherry-NCoA2^78^ and pmCherry-GR^11^ were a kind gift from Gordon Hager. pmCherry-GR^A477T^ (GRdim) was generated by using a QuikChange II XL Site Directed Mutagenesis Kit according to the manufacturer’s instructions (Stratagene, La Jolla, CA, USA).

### Cell culture and transient transfection

Newborn Hamster Kidney (BHK) cells were cultured in DMEM supplemented with 10% FBS plus penicillin (100 IU/ml) and streptomycin (100 µg/ml) at 37 °C under humidified atmosphere with 4.5% CO_2_. Transient transfections were performed with Lipofectamine 2000 according to manufacturer’s instructions. Briefly, 3 × 10^5^ cells were grown on coverslips, transfected with 1–1.5 µg of DNA and transfection medium was replaced with serum-free DMEM. Cells were incubated overnight with this medium prior to hormone stimulation and then incubated for at least 30 min with Dex (10 nM) or 21-OH (10 µM). Ethanol was used as vehicle for control experiments. For hormone washout experiments, cells were treated 1 h with Dex, washed with PBS and incubated in Dex-free medium for 20 h.

3617 cells were kindly provided by Gordon Hager and contain a tandem array (~200 copies) of a mouse mammary tumor virus long terminal repeat, Harvey viral ras (MMTV‑v‑Ha‑ras) reporter integrated into chromosome four^45^. Cells were routinely cultured in DMEM high glucose supplemented with 10% FBS (Life Technologies, Grand Island, NY, USA) and 2 mM L-glutamine (Life Technologies). In all cases, 3617 cells were grown in the presence of 5 µg/ml tetracycline (Sigma-Aldrich) to prevent expression of a stably integrated GFP-GR^11^. Transient transfections were performed with jetPRIME™ reagent (PolyPlus, New York, NY, USA) according to manufacturer’s instructions. Prior to hormone treatments cells were seeded to 2-well Lab-Tek chamber slides (Thermo Fisher, Waltham, MA, USA) and incubated overnight in DMEM containing 10% charcoal-stripped FBS and 2 mM L-glutamine. Hormone treatments were the same as in BHK cells.

### *In situ* nucleases digestion

BHK cells grown on coverslips, transfected and stimulated as previously described were fixed with 4% paraformaldehyde (PFA, Biopack, Buenos Aires, Argentina) in PBS for 5 min at room temperature, permeabilized with 100 mM NaCl, 300 mM sucrose, 10 mM Pipes pH 6.8, 3 mM MgCl_2_, 0.5% Triton X-100 for 10 min at 4 °C, incubated with extraction buffer (250 mM (NH_4_)_2_SO_4_, 300 mM sucrose, 10 mM Pipes pH 6.8, 3 mM MgCl_2_, 0.5% Triton X-100) for 5 min at 4 °C, digested with 50 mM NaCl, 300 mM sucrose, 10 mM Pipes pH 6.8, 3 mM MgCl_2_, 0.5% Triton X-100, 100 µg/ml DNase I or 100 µg/ml RNase A for 60 min at 37 °C, incubated again with extraction buffer for 5 min at 4 °C and finally fixed with 4% PFA in PBS for 15 min at room temperature.

### Confocal imaging

Confocal images were acquired in a FV1000 laser scanning microscope (Olympus), using an UPlanSApo 60x oil immersion objective (NA = 1.35). GFP and mCherry were excited using a multi-line Ar laser at 488 nm and a He-Ne green laser at 543 nm (average power at the sample, 700 nW), respectively. Fluorescence was detected with a photomultiplier set in the pseudo photon-counting detection mode. The pixel size was set at 83 nm.

Z-stack images of 3617 cells were acquired at the CRC, LRBGE Optical Imaging Core facility (NIH, Bethesda, Maryland, USA) in a LSM 780 laser scanning microscope (Carl Zeiss, Inc., Thornwood, NY, USA) equipped with an environmental chamber and a 40X oil immersion objective (NA = 1.4). The excitation source was a multi-line Ar laser tuned at 488 nm for GFP and a 594 nm laser for mCherry. Fluorescence was detected with a GaAsP detector in photon-counting mode using 490–561 nm and 606–695 nm filtering, respectively. Six z-planes separated in 1 µm were acquired from random fields of cells using a pixel size of 100 nm to rapidly find by eye the array of cells in the observed field. The fraction of cells showing a visible bright spot (signal/background >1.7) that corresponds to the MMTV-array (*p*_*array*_) was calculated as *p*_*array*_ = *N*_*array*_/*N*_*cells*_, where *N*_*cells*_ and *N*_*array*_ correspond to the total number of cells observed in each condition and the number of those cells presenting a visible array, respectively.

### Fluorescence Correlation Spectroscopy

Single-point FCS measurements were performed in an Olympus FV1000 confocal microscope set in the pseudo photon-counting mode at a fixed position of the nucleoplasm of cells presenting relative low fluorescence intensity (3 < signal/background < 30). The intensity was collected during ~3 min using a 20 µs sampling time. We avoid running multiple measurements in the same cell to minimize the photobleaching and photodamage of the observed cells.

Orbital scanning FCS was performed in a two-photon excitation microscope built around an Olympus IX70 body^79^ using a Chameleon Ultra Ti:Sapphire laser (Coherent) tuned at 810 nm and driven by SimFCS program (LFD, Irvine, CA, USA). The laser was scanned with two mirrors driven by a I/O Tec Card (Measurement Computing, USA). Fluorescence was collected with a 1.2-NA water objective (Olympus UplanSApo 60x) and split with a LP 550 nm dichroic mirror into two different channels to collect fluorescence with BP 505–535 nm and BP 602–678 nm filters.

The laser beam was set to repetitively trace orbits (radius = 2.6 µm) within the nucleus at a position selected by user. The total number of orbits was 6 × 10^5^. The pixel size and dwell time were 83 nm and 20 µs, respectively.

Line-scanning FCS experiments were run in the LSM 780 laser scanning microscope (Carl Zeiss, Inc., Thornwood, NY, USA) described above. Briefly, the laser was repetitively scanned in a 3.4 µm-line crossing the MMTV-array. The total number of lines was 2 × 10^5^. The pixel size, dwell time and line time were 70 nm, 6.82 µs and 0.764 ms, respectively.

### FCS analysis

ACFs and CCFs were calculated using SimFCS program (LFD, Irvine, CA, USA). In scanning FCS experiments, we used a tracking routine to locate the focus/array position from a kymograph^40^. Then, the integrated intensity at the focus/array position was calculated as a function of time and analyzed as described above.

### Foci analysis

Foci were identified in a central, optical slice of the nucleus using the density counting mode of *Ilastik* software designed to count objects in crowded scenarios (www.ilastik.org)^80^. Briefly, the user manually identifies the background and 5–10 foci which are then used as learning-patterns to identify the rest of the foci. The *Ilastik* image generated in this analysis was binarized and processed using the “Analyze Particles” tool of ImageJ (NIH, USA) to count foci and measure their intensity. The nuclear area was recovered from the original image with ImageJ. Foci colocalization was estimated with an object-based method described in ref. 81. We assumed that different-color foci colocalize if they are within 0.2 µm (i.e. the optical resolution) from each other. The percentage of colocalized foci was calculated respect to the channel with lower foci density.

### Calculation of the apparent association constant (K_a_)

The concentration of GR, NCoA2 and GR/NCoA-2 complex were calculated from ACFs and CCF amplitudes as previously described^82^:

1 $$\begin{array}{rcl}\quad \,\,\,{[GR]}_{Free} & = & \frac{\gamma }{{G}_{GR}\cdot {N}_{A}\cdot {V}_{PSF}}-[Complex],\\ {[NCoA2]}_{Free} & = & \frac{\gamma }{{G}_{NCoA2}\cdot {N}_{A}\cdot {V}_{PSF}}-[Complex],\\ \,\,\,[Complex] & = & \,\frac{\gamma \cdot {G}_{cc}}{{N}_{A}\cdot {V}_{PSF}\cdot {G}_{GR}\cdot {G}_{NCoA2}}\end{array}$$

where γ = 0.35 is a geometric factor that depends on detection profile^83^, V_PSF_ = ω_r_^2^. ω_z_. (π/2)^3/2^ is the volume of the point spread function (*PSF*), N_A_ is the Avogadro number and G_GR_, G_NCoA2_ and G_cc_ are the amplitudes of GR channel, NCoA-2 channel and CCF, respectively. ω_r_ = 0.25 µm and ω_z_ = 1 µm are the radial and axial waist of the *PSF*, respectively. K_a_ was calculated assuming a 1:1 stoichiometry.

### Quantitative analysis of FCS data

ACF data from FCS experiments was analyzed using an extended version of the reaction dominant model described in ref. 41 that considers binding to two populations of fixed sites^35^:

2 $$G(\tau )=\frac{1}{{2}^{3/2}N}[{f}_{D}{(1+\frac{\tau }{{\tau }_{D}})}^{-1}{(1+\frac{\tau }{{\omega }^{2}{\tau }_{D}})}^{-1/2}+{f}_{short}{e}^{-\tau /{\tau }_{short}}+{f}_{long}{e}^{-\tau /{\tau }_{long}}]$$

where *N* is the mean number of fluorescent molecules in the confocal volume, τ_D_ is the characteristic diffusion time, ω is the ratio between axial and radial waists of the observation volume, and *f*_*D*_ is the freely diffusing population fraction. *f*_*short*_ and *f*_*long*_ are the population fractions bound to shorter-lived and longer-lived targets, and *τ*_*short*_ and *τ*_*long*_ are their residence times. The reciprocal of the residence time corresponds to the dissociation constant k_off_.

The model does not consider the intranuclear variability in affinities that may be present in longer-lived and shorter-lived fractions^84^ only providing mean effective values for these parameters. On the other hand, this equation assumes a large and constant concentration of binding sites that are independent from each other^41^.

ACF curves were fitted with equation (2) applying a global routine and assuming a common residence time (longer- and shorter-lived) for each experimental condition^35^ with the exception of scanning-FCS experiments that showed a higher cell-to-cell variability.

ACF data obtained from line-scanning FCS experiments were analyzed with the following equation:

3 $$G(\tau )=\frac{1}{{2}^{3/2}N}[{p}_{short}{e}^{-\tau /{\tau }_{short}}+{p}_{long}{e}^{-\tau /{\tau }_{long}}]$$

where *p*_*short*_ and *p*_*long*_ are the fraction of bound molecules attached to either shorter-lived or longer-lived targets, respectively. This expression considers that the characteristic diffusion time is smaller than the sampling time.

### Statistical analysis

Results were expressed as means ± SEM. Statistical analyses were performed with STATISTICA 7.0 (StatSoft, Inc.) and consisted of one-way ANOVA followed by Tukey’s or Dunnett’s tests. Before statistical analysis, data were tested for homogeneity of variances using Levene’s test. Student’s t-test was performed for individual pairwise comparisons. Differences were regarded as significant at p < 0.05.

Comparisons of p_array_ in different conditions were done using the Marascuilo’s multiple comparisons procedure^85^. The test proceeds in two different steps and depends on a critical value related to the sample sizes and a chi-squared distribution. First, the frequencies *p*_*array*,*i*_ (where *i*, is the experimental conditions) are compared using a chi-square test of homogeneity to determine whether frequency counts are distributed identically across the different *i* conditions. The rejection of the null hypothesis (i.e. the frequency is the same for all the conditions) implies that the *p*_*array*,*i*_ frequency is different in at least one of the conditions.

Then, the test compares the absolute difference between two frequencies *p*_*array*,*i*_ − *p*_*array*,*j*_ with a critical range (*r*_*ij*_):

4 $${r}_{ij}=\,\sqrt{{\chi }_{1-\alpha ,k-1}^{2}}\sqrt{\frac{{p}_{array,i}(1-{p}_{array,i})}{{n}_{i}}+\frac{{p}_{array,j}(1-{p}_{array,j})}{{n}_{j}}}$$

where *n*_*i*_ and *n*_*j*_ are the sample sizes, *k* is the total number of data sets and ${\chi }_{1-\alpha ,k-1}^{2}$ is the critical value of the chi-square distribution, for a significance level of *α* and *k*-1 degrees of freedom.

Frequencies *p*_*array*,*i*_ and *p*_*array*, *j*_ are considered significantly different when *p*_*array*,*i*_ − *p*_*array*,*j*_ > *r*_*ij*_.

## Electronic supplementary material

Supplementary Information
