# Supplementary material for: Mapping the Dynamics of the Glucocorticoid Receptor within the Nuclear Landscape
Source: Sci Rep. 2017 Jul 24;7:6219. doi: 10.1038/s41598-017-06676-0 (PMC5524710; doi:10.1038/s41598-017-06676-0)
Supplement: Supplementary file 1 — Supplementary Information [file 41598_2017_6676_MOESM1_ESM.pdf]

Supplementary Information

Mapping the Dynamics of the Glucocorticoid Receptor within the Nuclear Landscape

Martin Stortz, Diego M. Presman, Luciana Bruno, Paolo Annibale, Maria V. Dansey, Gerardo Burton, Enrico Gratton, Adali Pecci* and Valeria Levi*

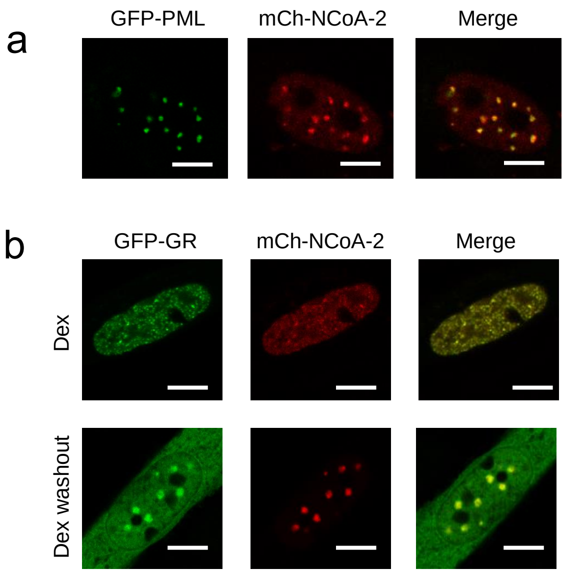

Supplementary Figure S1

Characterization of NCoA-2 bodies. (a) Representative images of a BHK cell co-expressing GFP-PML(I) and mCherry-NCoA-2 (Scale bar: 5 µm). **(b)** Representative images of BHK cells co-expressing GFP-GR and mCherryNCoA-2 and stimulated 1 h with Dex (top panels). After this incubation, cells were washed with PBS and incubated in Dex-free medium for 20 h (bottom panels) (Scale bar: 5 µm).

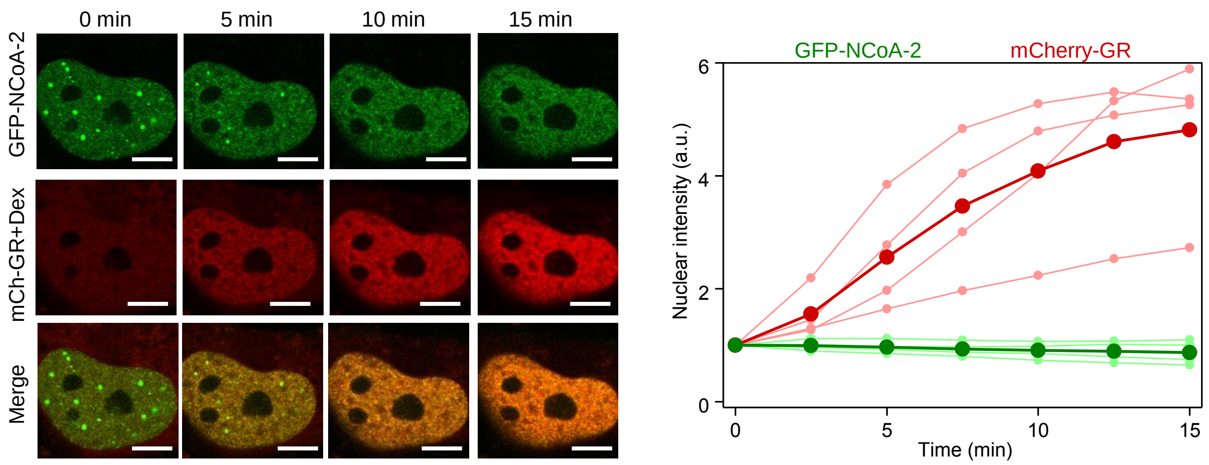

Supplementary Figure S2

Time-lapsed imaging of GR and NCoA-2 upon hormone addition. Representative images of a BHK cell co-expressing GFP-NCoA-2 and mCherry-GR, during the first 15 min after Dex-stimulation (Scale bar: 5 µm). Right panel shows the time evolution of the relative mean nuclear intensity of GFP-NCoA-2 (green lines) and mCherry-GR (red lines). Light curves represent single-cell values, while dark curves represent mean values.

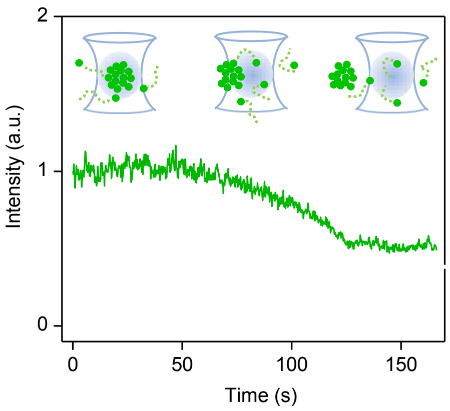

Supplementary Figure S3

GR focus motion during single-point FCS measurements. Relative fluorescence intensity time-trace from a single-point FCS measurement performed on a focus in Dex-stimulated BHK cells expressing GFP-GR.

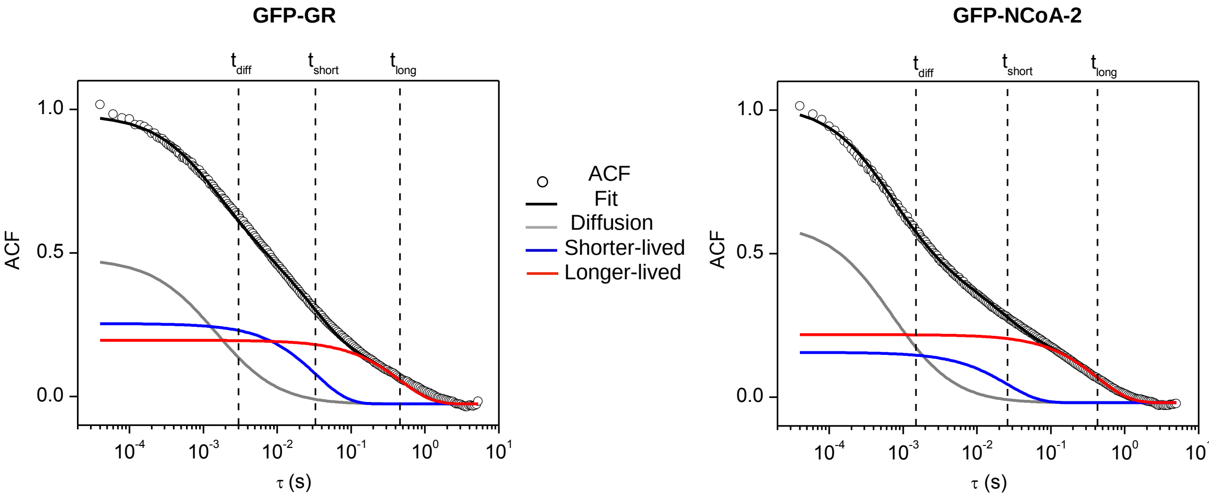

Supplementary Figure S4

FCS analysis of GR and NCoA-2 dynamics in the nucleus. Average ACF data of GFP-GR (left panel) or GFP-NCoA-2 (right panel) in cells stimulated with Dex fitted with a model considering diffusion and binding to fixed targets with different residence times (continuous lines). The dashed vertical lines show the characteristic diffusion time and the residence times at shorter-lived and longer-lived sites.

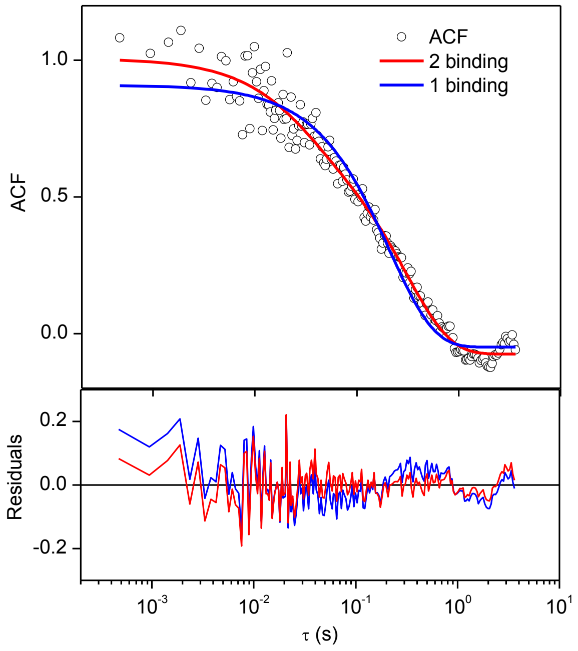

Supplementary Figure S5

GR dynamics at the MMTV-array. The average ACF data of GFP-GR in 3617 cells stimulated with Dex obtained from line-scanning FCS experiments on the MMTV-array was fitted with a model considering a single or two populations of fixed binding sites.

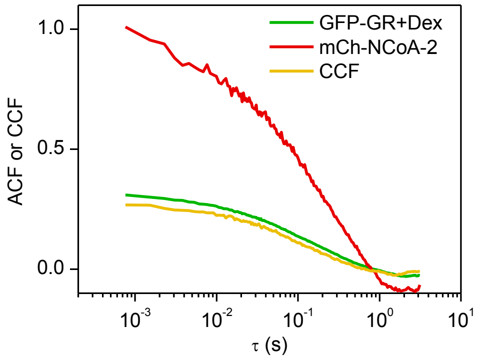

Supplementary Figure S6

ACFs and CCF analysis of GR and NCoA-2 on the MMTV-array. Average ACF and CCF data of GFP-GR and mCherry-NCoA-2 in 3617 cells stimulated with Dex. The data were normalized to the ACF amplitude of mCherry-NCoA-2.

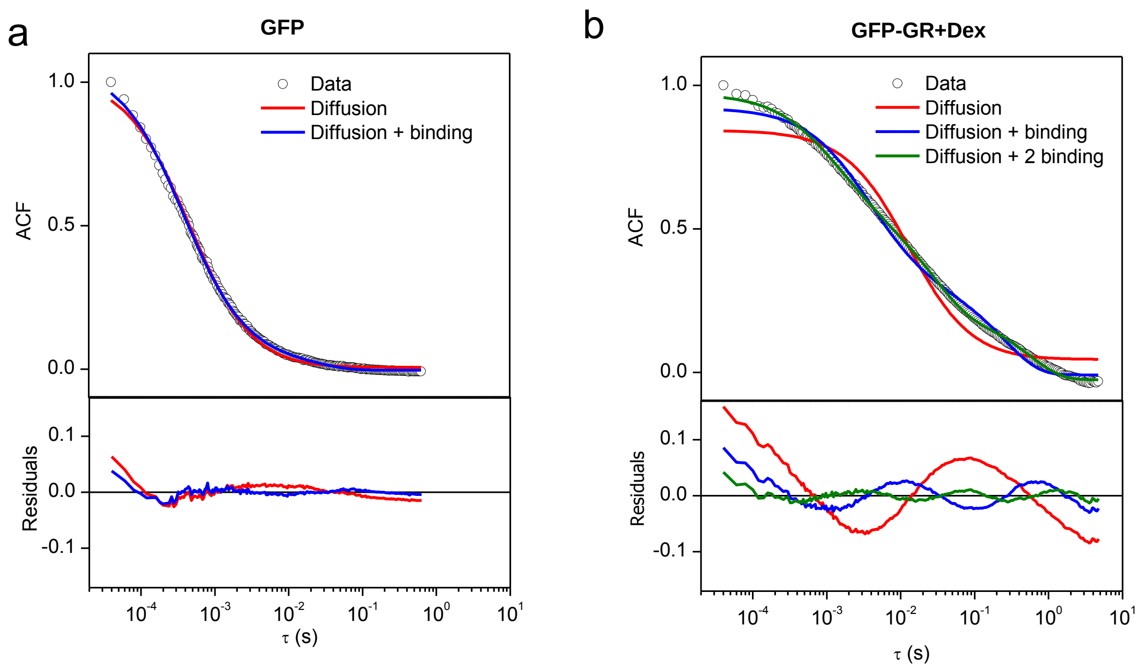

Supplementary Figure S7

GFP and GR-GFP mobility in the nucleus. Average ACF data of GFP **(a)** or GFP-GR activated by Dex **(b)** in the nucleus of BHK cells was fitted with models that considers Brownian diffusion (red lines) or diffusion and binding to a single (blue lines) or two (green line) populations of binding sites.

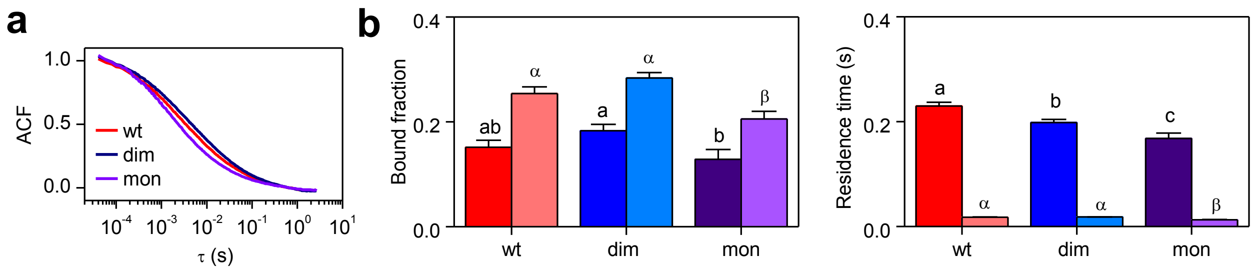

Supplementary Figure S8

Fluorescence correlation spectroscopy analysis of GR dynamics in control cells. **(a)** Average normalized ACFs of the receptor (wild type, GRdim or GRmon) fused to GFP in non-stimulated BHK cells. **(b)** Parameters obtained by fitting equation (2) corresponding to the short-lived (light colors) and long-lived (dark colors) components ($22 \leq n \leq 32$). Bars corresponding to the same variable with different superscript letters are significantly different from each other ($p<0.05$).

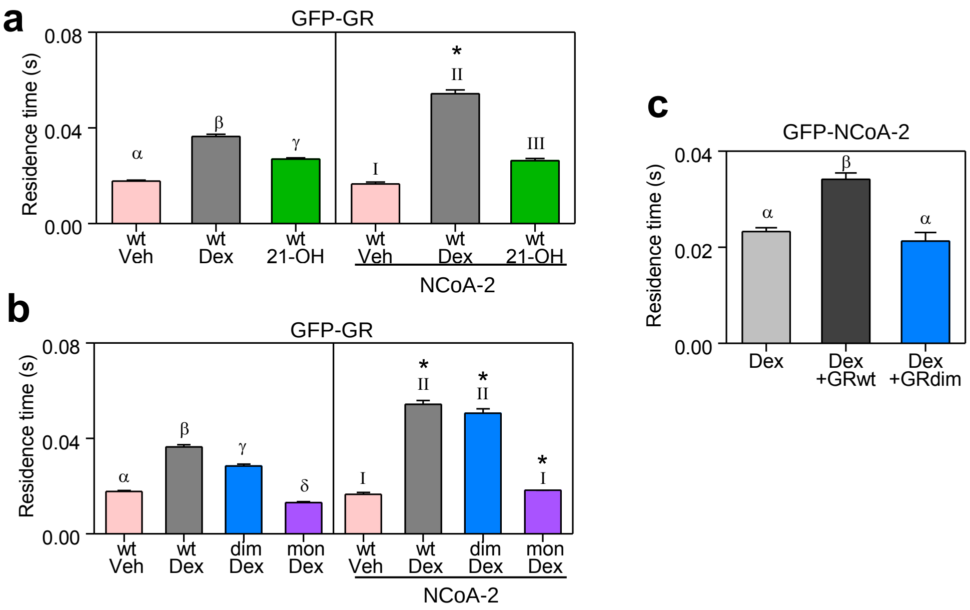

Supplementary Figure S9

Residence times of GR and NCoA-2 at shorter-lived sites. Single-point FCS measurements were run in the nucleoplasm of BHK living cells expressing GFP fused to **(a-b)** the wild type GR, GRdim or GRmon and treated with Dex, 21-OH or vehicle (Veh), in the absence or presence of mCherry-NCoA-2, or **(c)** NCoA-2 and treated with Dex, in the absence or presence of mCherry-GR or mCherry-GRdim. **(a-c)** Residence times of the short-lived component were obtained by fitting equation (2) ($22 < n < 43$). (**a-b)** Asterisks (*) indicate the residence time significantly differs due to mCherry-NCoA-2 overxpression ($p<0.05$). Greek letters (α, β, γ, δ) or Roman numbers (I, II, III) indicate significant differences in the residence time measured in the absence or prescence of mCherry-NCoA-2, respectively ($p<0.05$). **(c)** Bars with different supperscript letters are significantly different from each other ($p<0.05$).
